# Analytical cohort study on intraoperative hypotension as a risk factor for postoperative acute kidney injury

**DOI:** 10.6026/973206300213223

**Published:** 2025-09-30

**Authors:** Vishal Mruthyunjaya Kalmani, Dhyaneshwar Ra, Yeruva Bheemeswara Reddy, Poornima Venkatraman

**Affiliations:** 1 Department of Surgery, Barking Havering and Redbridge University Hospitals NHS Trust, Essex, United Kingdom; 2 Department of Emergency of General Surgery, Government Stanley Medical College Chennai, Tamil Nadu, India; 3 Department of Surgery, Santhiram Medical College, Andhra Pradesh, India; 4 Department of Surgery, Davao Medical School Foundation, Philippines

**Keywords:** Mean arterial pressure, acute renal damage, perioperative risk, analytical cohort studies, intraoperative hypotension

## Abstract

Acute kidney injury (AKI) is a serious complication that happens after surgery and intraoperative hypotension (IOH) is a modifiable risk factor. We did a cohort analytical study consisting of 145 adult patients who were undergoing major non-cardiac surgery under general anesthesia. Patients who were diagnosed with IOH (defined as mean arterial pressure <65 mmHg for ≥15 minutes), had significantly higher rates of postoperative AKI. Multivariate analysis confirmed independent predictors of AKI including IOH, chronic kidney disease, and prolonged operative time. Overall, this study highlights the importance of hemodynamic monitoring and the continuing maintenance of MAP ≥65 mmHg as a potential way to reduce renal complications.

## Background:

Acute kidney injury (AKI) is a frequent adverse outcome following liver transplantation (LT) with a multifactorial etiology. It is critical to identify modifiable risk factors to mitigate the risk [1]. Acute kidney injury (AKI) is a frequent and dangerous side effect of surgery that raises mortality, morbidity and medical expenses [2]. There is no consensus regarding safe intraoperative blood pressure thresholds that protect against postoperative acute kidney injury (AKI) [3]. One of the many perioperative risk factors linked to AKI that has drawn increased attention recently is intraoperative hypotension (IOH), which has a propensity to encourage ischemia damage and renal hypoperfusion [4]. Although anesthetic and hemodynamic monitoring technology has advanced, intraoperative hemorrhage (IOH) remains a common surgical operation that is either misdiagnosed or treated insufficiently [5]. New evidence showed IOH does not increase risk of postoperative mobility. Intraoperative hypotension (IOH) or highly invasive surgery adversely affects postoperative clinical outcomes [6]. It is, however, unclear whether IOH affects postoperative acute kidney injury (AKI) depending on the invasiveness of abdominal surgery [7]. Recent studies show a direct correlation between the onset of postoperative AKI and the length and severity of IOH, especially when MAP drops below key thresholds like 65 mmHg [8]. Nevertheless, because surgical risk profiles differ, patient populations are diverse and definitions of IOH differ, it is challenging to establish direct causation [9]. The kidneys' ability to autoregulate perfusion is restricted due to their high vascularity [10]. Persistent hypotension that interferes with this autoregulation raises the risk of AKI, especially in patients who already have renal insufficiency or who have diabetes and hypertension [11]. Since IOH can be prevented, knowing its role in the pathogenesis of AKI is crucial for guiding intraoperative treatment approaches [12]. In a diverse surgical group, this analytical cohort study examines the connection between IOH and postoperative AKI using time-based hypotension criteria and accepted definitions [13]. Therefore, it is of interest to reduce renal consequences and promote evidence-based preoperative care by identifying IOH as a modifiable risk factor.

## Materials and Methods:

The 145 adult patients who had major non-cardiac surgery performed under general anesthesia between January 2021 and December 2023 were included in this analytical cohort study, which was carried out at a tertiary care facility. Individuals requiring urgent surgery, those with pre-existing end-stage renal illness and those with insufficient intraoperative hemodynamic data were not included. Intraoperative hypotension (IOH) was defined as a mean arterial pressure (MAP) <65 mmHg maintained for ≥15 cumulative minutes during surgery. Intraoperative blood pressure readings were taken using either invasive or non-invasive monitoring at one-minute intervals. According to the Kidney Disease: Improving Global Outcomes (KDIGO) criteria, which include a rise in serum creatinine of at least 0.3 mg/dL within 48 hours or a rise of at least 50% from baseline, postoperative acute kidney injury (AKI) was detected within 48 hours. Patients were stratified into two cohorts: those who experienced IOH and those who did not. Relevant perioperative variables, including demographics, comorbidities (such as diabetes, hypertension and chronic kidney disease), type and duration of surgery, baseline renal function, fluid balance and use of nephrotoxic agents, were collected from anesthesia and surgical records. Statistical analysis was performed using SPSS version 26. Continuous variables were compared using independent t-tests or Mann-Whitney U tests, while categorical variables were analyzed using chi-square or Fisher's exact tests. Multivariate logistic regression was used to identify independent predictors of postoperative AKI, adjusting for potential confounders. A p-value <0.05 was considered statistically significant.

## Results:

Of the 145 patients included in the study, 58 (40%) experienced intraoperative hypotension (IOH), defined as MAP <65 mmHg for ≥15 minutes. Postoperative acute kidney injury (AKI) developed in 32 patients (22%), with a significantly higher incidence in those with IOH. Multivariate analysis confirmed IOH as an independent predictor of AKI, along with pre-existing chronic kidney disease (CKD) and prolonged operative time. Table 1 (see PDF) shows patients with intraoperative hypotension were slightly older and had higher rates of baseline CKD and diabetes. Table 2 (see PDF) shows baseline renal parameters were significantly worse in the IOH group. Table 3 (see PDF) shows IOH was most common in abdominal and vascular surgeries and surgery duration was significantly longer. Table 4 (see PDF) shows incidence of postoperative AKI was significantly higher in the IOH group. Table 5 (see PDF) shows among patients with AKI, most had Stage 1 injury, but progression to Stage 2 was more common in the IOH group. Table 6 (see PDF) shows patients with IOH received more intraoperative fluids and vasopressors. Table 7 (see PDF) shows postoperative serum creatinine rise was more marked in the IOH group. Table 8 (see PDF) shows ICU admission and prolonged hospital stays were more common in patients who experienced IOH. Table 9 (see PDF) shows multivariate regression identified IOH as an independent predictor of postoperative AKI. Table 10 (see PDF) shows the majority of patients with IOH and AKI did not receive nephrology consultation, indicating a gap in early management.

## Discussion:

This analytical cohort study provides strong evidence that intraoperative hypotension (IOH), particularly when MAP falls below 65 mmHg for a cumulative duration of 15 minutes or more, is a significant and independent predictor of postoperative acute kidney injury (AKI). Patients experiencing IOH had a higher incidence of AKI, greater creatinine rise, longer hospital stays and higher rates of ICU admission and 30-day readmissions [14]. These findings are consistent with previous studies suggesting that renal perfusion is highly sensitive to even brief periods of low MAP during surgery [15]. The association remained significant even after controlling for confounding factors such as pre-existing chronic kidney disease (CKD), operative duration and diabetes mellitus. Interestingly, a large proportion of patients with AKI did not receive early nephrology consultation, highlighting a missed opportunity for timely intervention [16]. Furthermore, the lack of routine intraoperative urine output monitoring in many cases indicates insufficient real-time renal risk assessment. The findings emphasize the importance of maintaining MAP above 65 mmHg during surgery, especially in high-risk individuals [17]. Intraoperative strategies such as vigilant hemodynamic monitoring, early use of vasopressors, judicious fluid administration and avoidance of nephrotoxic agents can help mitigate this risk. Additionally, incorporating real-time decision support systems and predefined MAP targets into anesthetic protocols could improve patient outcomes [18]. Limitations of this study include its single-center, retrospective design and lack of long-term renal outcome data. However, its strength lies in the consistent definition of IOH, use of standardized KDIGO criteria for AKI diagnosis and comprehensive data analysis. Future prospective studies with real-time hemodynamic interventions are warranted to establish causality and develop actionable clinical pathways. Overall this study underscores the critical need for intraoperative hemodynamic vigilance and supports the integration of renal protective strategies into perioperative care, especially for patients at elevated baseline risk.

## Conclusion:

Intraoperative hypotension is an independent risk factor for postoperative acute kidney injury in major non-cardiac surgeries. Maintaining MAP ≥65 mmHg during surgery can substantially reduce the incidence of AKI and associated complications. Perioperative protocols must prioritize hemodynamic stability to protect renal function and improve surgical outcomes.
